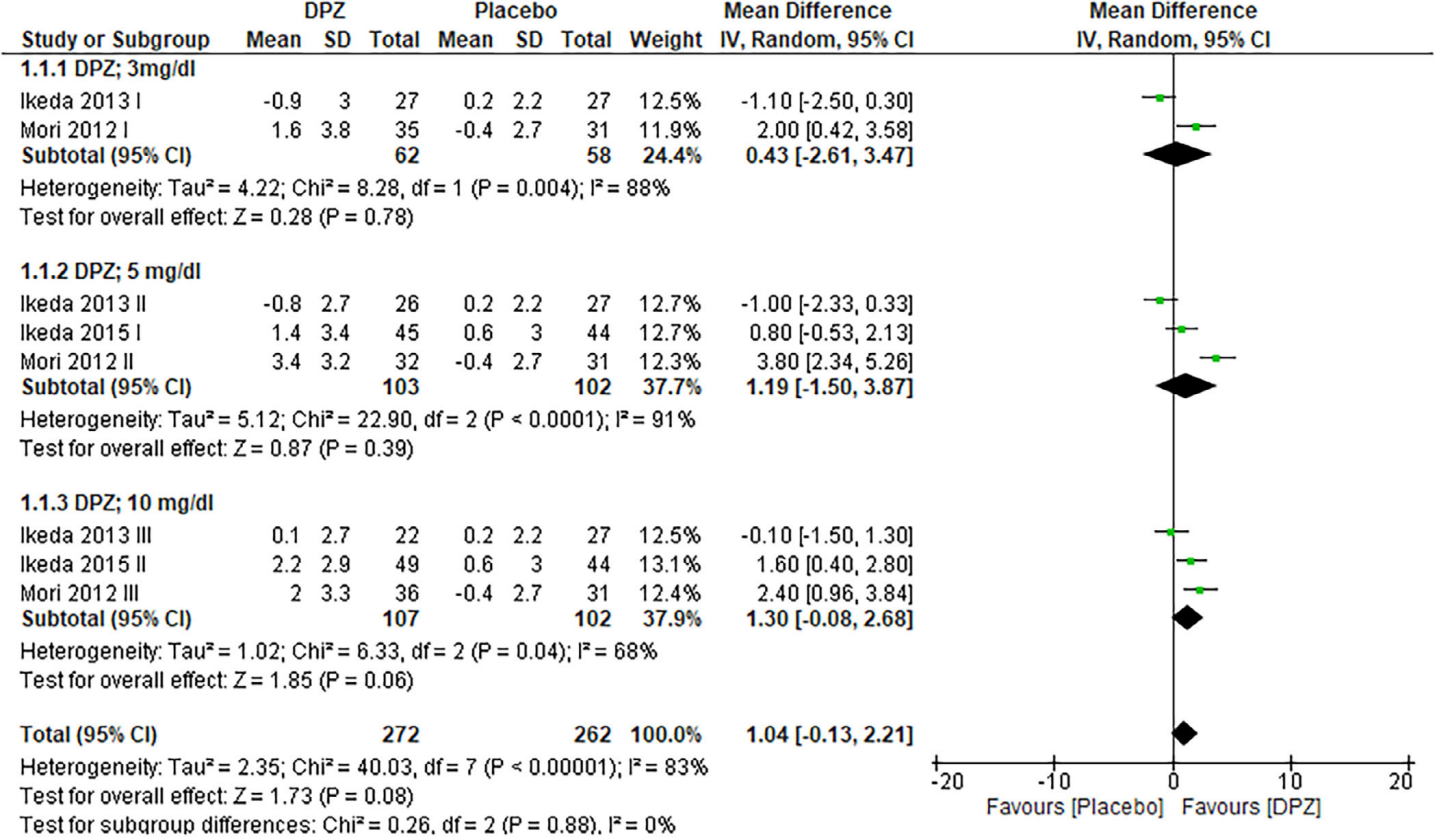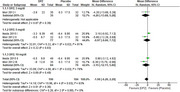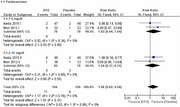# Efficacy and Safety of Donepezil for Lewy Body Dementia: A Systematic Review and Meta‐Analysis

**DOI:** 10.1002/alz70859_103155

**Published:** 2025-12-25

**Authors:** Noreen M. El‐Bayaa, Nada A. Hamdan, Mariam M. Fouad, Nadeen A Nagy, Youssef A. Ismail

**Affiliations:** ^1^ Faculty of Medicine Port Said Univeristy, Egypt, Port Said, Port Said Egypt

## Abstract

**Background:**

Lewy Body Dementia (LBD) is a frequent form of dementia that occurs when protein clusters known as Lewy bodies accumulate in the brain. This study aims to investigate the efficacy and safety of Donepezil to improve cognitive and behavioral symptoms in people living with Lewy Body Dementia.

**Methods:**

We systematically searched in Cochrane, PubMed, Web of Science and SCOPUS since date of inception until Dec 1^st^ 2024. Meta‐analyses were performed using random effects models to calculate pooled mean differences.

**Results:**

We initially identified 968 studies through database search and included only four studies in the systematic review, three of them were included in the meta‐analysis. Included studies showed a total of 453 participants (269 females, 59.38 %), 369 participants received the drug and 84 received placebo. Regarding 3 mg/d, the overall MMSE mean difference between donepezil and placebo did not favor either of the groups (pooled MD = 0.43, 95% CI [‐2.61, 3.47], P = 0.78), regarding 5 mg/d, the overall mean difference also did not favor either of the groups (pooled MD = 1.19, 95% CI [‐1.50, 3.87], P = 0.39), and regarding 10 mg/d, the overall mean difference also did not favor either of the groups (pooled MD = 1.30, 95% CI [‐0.08, 2.68], P = 0.06). Neuropsychiatric Inventory (NPI‐10) scores showed no significant differences between donepezil and placebo groups across all doses. Similar results were also observed for NPI‐2 and ZBI scores. Regarding 5 mg/d, the pooled risk ratio for parkinsonism between donepezil and placebo did not favor either of the groups (pooled RR = 1.83, 95% CI [0.44, 7.64], P = 0.40 and regarding 10 mg/d, the pooled risk ratio also did not favor either of the groups (pooled RR = 1.54, 95% CI [0.37, 6.31], P = 0.55).

**Conclusion:**

This meta‐analysis of randomized controlled trials did not find significant differences in cognitive function (MMSE) or behavioral symptoms (NPI‐10, NPI‐2, ZBI) between donepezil (3, 5, and 10 mg/day) and placebo in patients with LBD.